# Supplementary material for: Structural Characterization and Assessment of Anti-Inflammatory and Anti-Tyrosinase Activities of Polyphenols from Melastoma normale
Source: Molecules. 2021 Jun 26;26(13):3913. doi: 10.3390/molecules26133913 (PMC8271706; doi:10.3390/molecules26133913)
Supplement: Supplementary file 1 [file molecules-26-03913-s001.zip › molecules-1239853-supplementary.pdf]

Supplementary Materials

Structural characterization and assessment of the tyrosinase activity of polyphenols from *Melastoma normale*

Rui-jie He [1, 2, 3], Jun Li [3], Yong-Lin Huang [2, *], Ya-Feng Wang [2], Bing-yuan Yang [2], Zhang-bin Liu [2], Li Ge [1], Ke-di Yang [1, *], **Dian-Peng Li [1, 2, *]**

[1] School of Chemistry and Chemical Engineering, Guangxi University, Nanning 530004, China ; hrj937@gxib.cn (R.-J.H.); 49745187@qq.com (L.G.); kdyang@163.com; ldp@gxib.cn (D.-P.L.)

[2] Guangxi Key Laboratory of Functional Phytochemicals Research and Utilization, Guangxi Institute of Botany, Guangxi Zhuang Autonomous Region and Chinese Academy of Sciences, Guilin 541006, China; hyl@gxib.cn (Y.-L.H.); wyf@gxib.cn (Y.-F.W.); yby@gxib.cn (Y.-B.Y.); 1543153384@qq.com (Z.-B.L.)

[3] State Key Laboratory for the Chemistry and Molecular Engineering of Medicinal Resources, College of Chemistry and Pharmacy, Guangxi Normal University, Guilin 541004, China; lijun9593@gxnu.edu.cn (J.L.)

* Correspondence: hyl@gxib.cn; Tel.: +86-773-355-0829; Fax: +86-773-355-0067

Figure S1. ^{1}H NMR (500 MHz, Methanol-d_4) spectrum of **1**
Figure S2. ^{13}C NMR (125 MHz, Methanol-d_4) spectrum of **1**
Figure S3. HSQC spectrum of **1** in Methanol-d_4
Figure S4. HMBC spectrum of **1** in Methanol-d_4
Figure S5. ^{1}H-^{1}H COSY spectrum of **1** in Methanol-d_4
Figure S6. Roesy spectrum of **1** in Methanol-d_4
Figure S7. HRESIMS spectrum of **1**
Figure S8. ^{1}H NMR (500 MHz, Methanol-d_4) spectrum of **2**
Figure S9. DEPTQ (125 MHz, Methanol-d_4) spectrum of **2**
Figure S10. HSQC spectrum of **2** in Methanol-d_4
Figure S11. HMBC spectrum of **2** in Methanol-d_4
Figure S12. ^{1}H-^{1}H COSY spectrum of **2** in Methanol-d_4
Figure S13. HRESIMS spectrum of **2**
Figure S14. ^{1}H NMR (500 MHz, Methanol-d_4) spectrum of **3**
Figure S15. DEPTQ (125 MHz, Methanol-d_4) spectrum of **3**
Figure S16. HSQC spectrum of **3** in Methanol-d_4
Figure S17. HMBC spectrum of **3** in Methanol-d_4
Figure S18. ^{1}H-^{1}H COSY spectrum of **3** in Methanol-d_4
Figure S19. HRESIMS spectrum of **3**
Figure S20. ^{1}H NMR (500 MHz, Methanol-d_4) spectrum of **4**
Figure S21. DEPTQ (125 MHz, Methanol-d_4) spectrum of **4**
Figure S22. HSQC spectrum of **4** in Methanol-d_4
Figure S23. HMBC spectrum of **4** in Methanol-d_4
Figure S24. ^{1}H-^{1}H COSY spectrum of **4** in Methanol-d_4
Figure S25. HRESIMS spectrum of **4**
Figure S26. CD spectrum of **1**— **4**

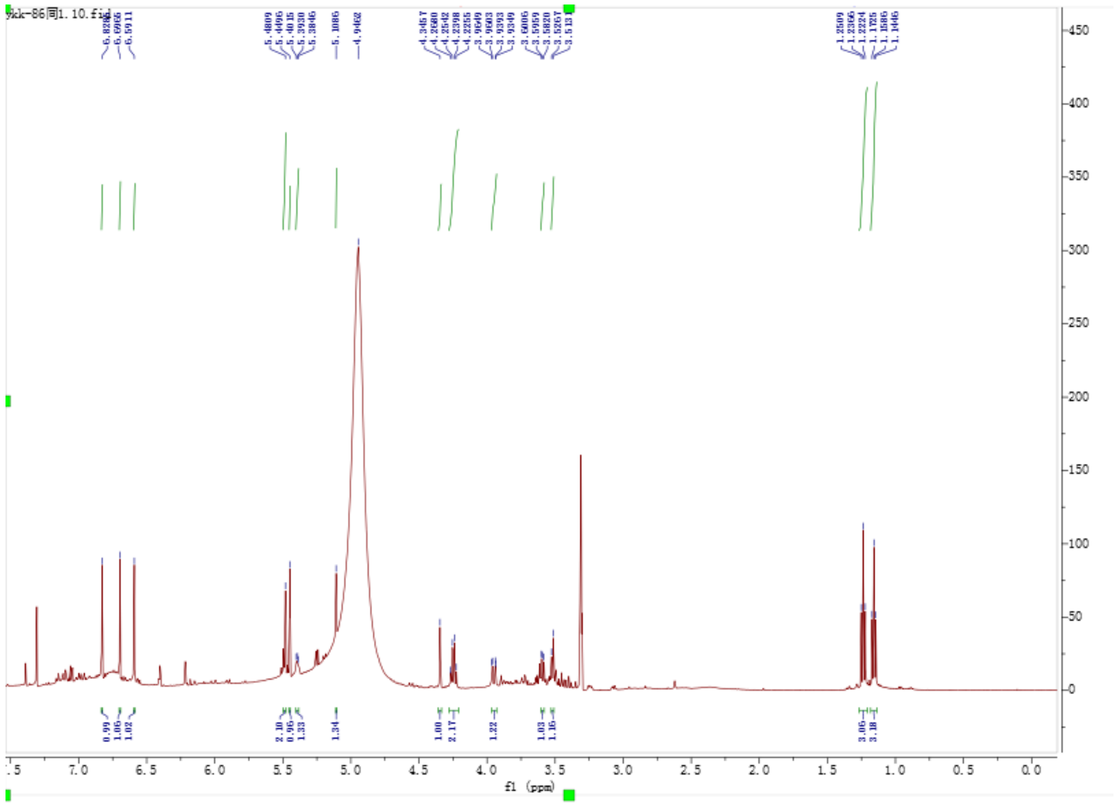

Figure S1. ^{1}H NMR (500 MHz, Methanol-d_4) spectrum of **1**

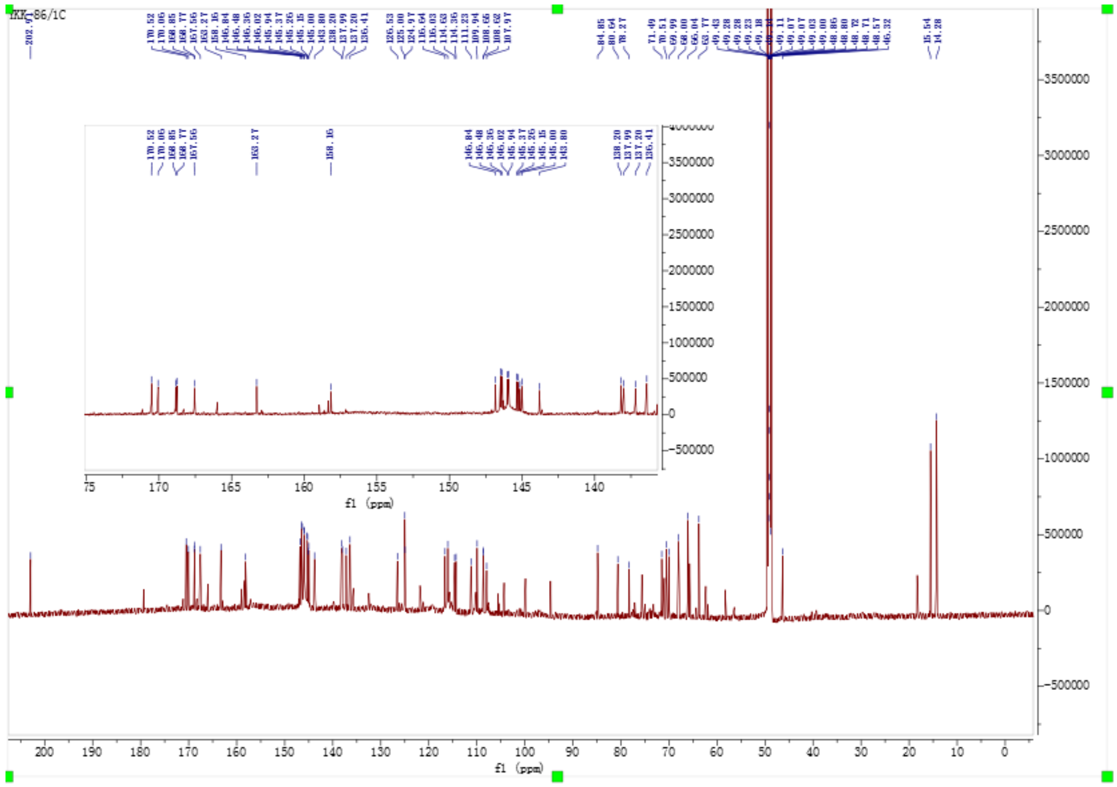

Figure S2. ^{13}C NMR (125 MHz, Methanol-d_4) spectrum of **1**

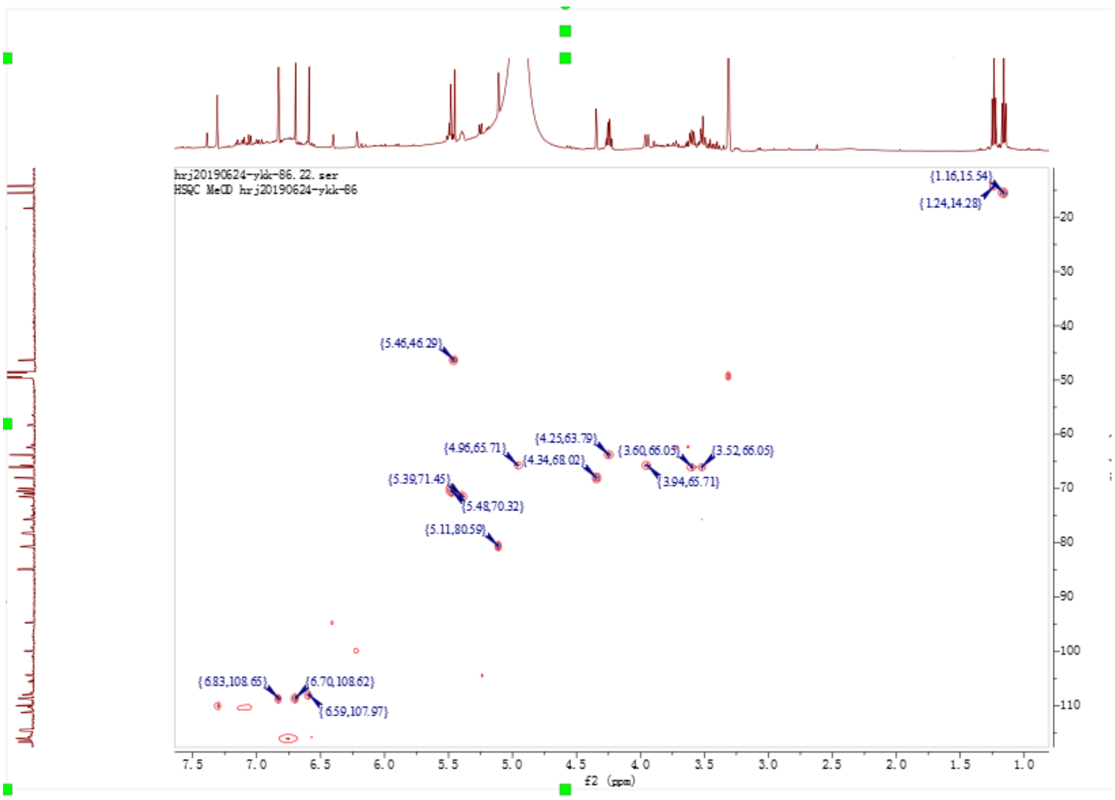

Figure S3. HSQC spectrum of **1** in Methanol-d_4

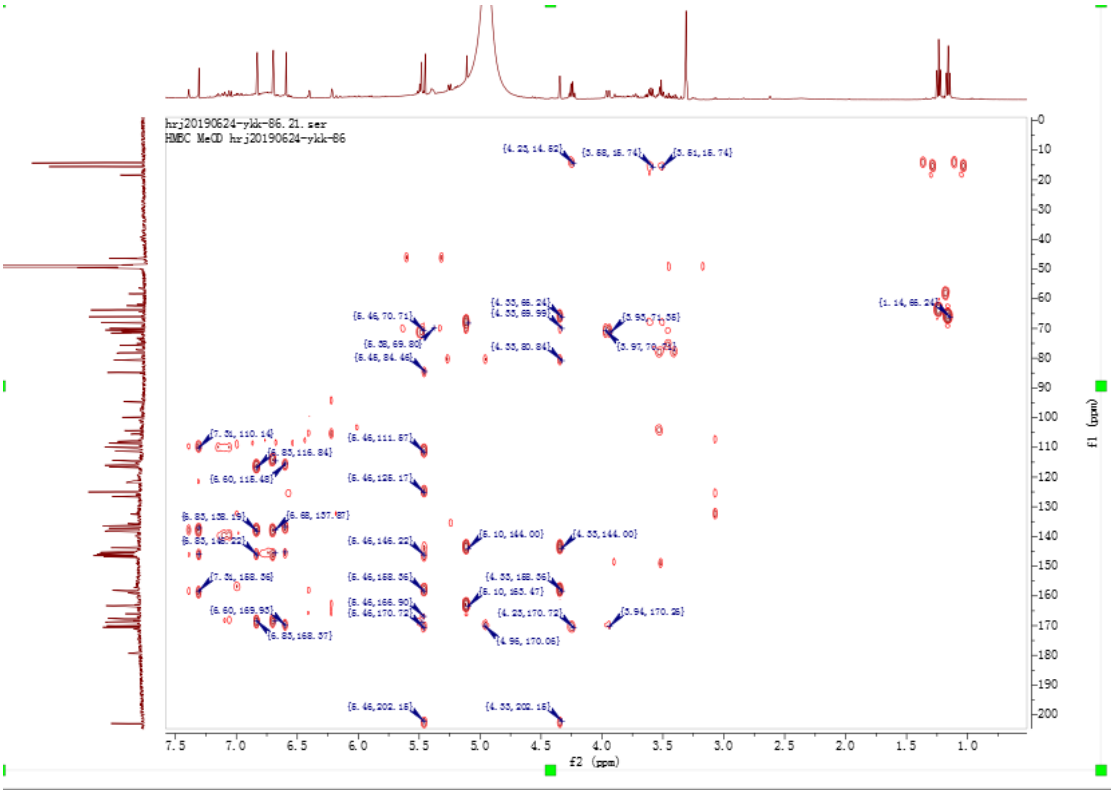

Figure S4. HMBC spectrum of **1** in Methanol-d_4

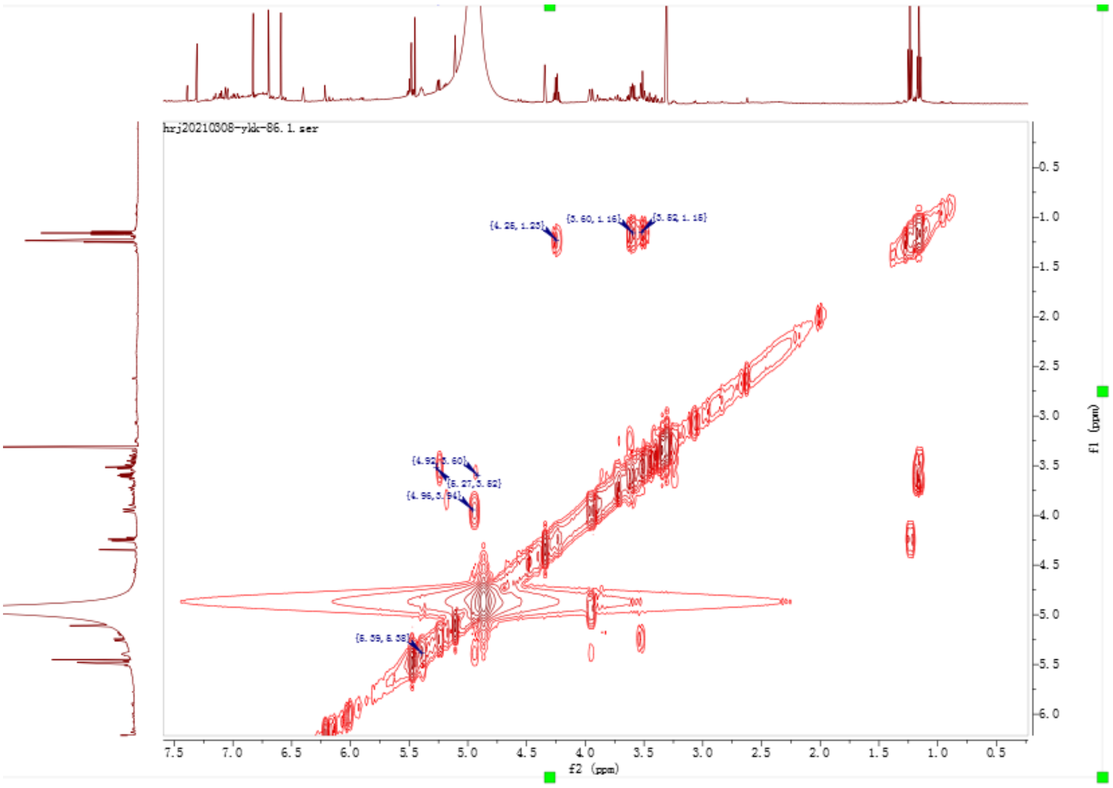

Figure S5. ^{1}H-^{1}H COSY spectrum of **1** in Methanol-d_4

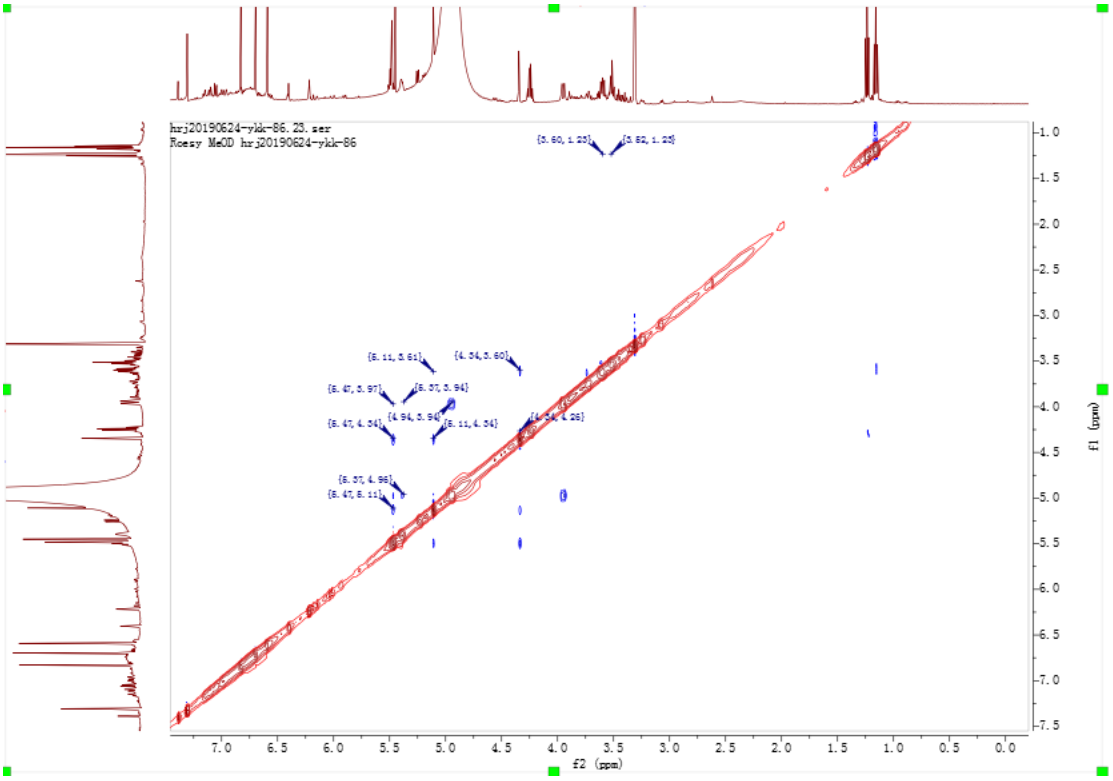

Figure S6. Roesy spectrum of **1** in Methanol-d_4

Elmt	Val.	Min	Max
H	1	1	100
C	4	1	100
N	3	0	0

Elmt	Val.	Min	Max
O	2	1	100
S	2	0	0

Use Adduct
H

Error Margin (ppm): 50
HC Ratio: 0.1 - 4.0
Max Isotopes: all
MSn Iso RI (%): 75.00

DBE Range: 1.0 - 50.0
Apply N Rule: no
Isotope RI (%): 1.00
MSn Logic Mode: AND

Electron Ions: both
Use MSn Info: no
Isotope Res: 10000
Max Results: 500

Event#: 4 MS(E-) Ret. Time : 22.277 Scan# : 2552

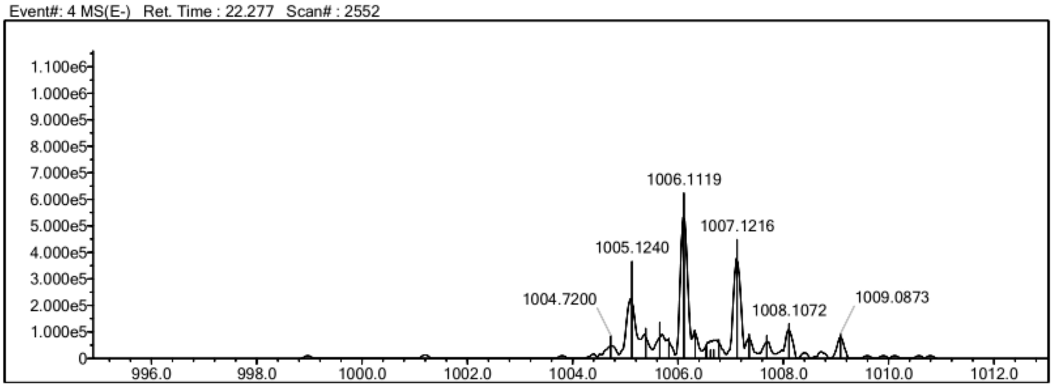

Rank	Score	Formula (M)	Ion	Meas. m/z	Pred. m/z	DBE	Df. (ppm)	Iso	Meas. (M)
2	18.75	C45 H34 O27	[M-H]-	1005.1240	1005.1215	29.0	2.49	19.48	1006.1313

Figure S7. HRESIMS spectrum of **1**

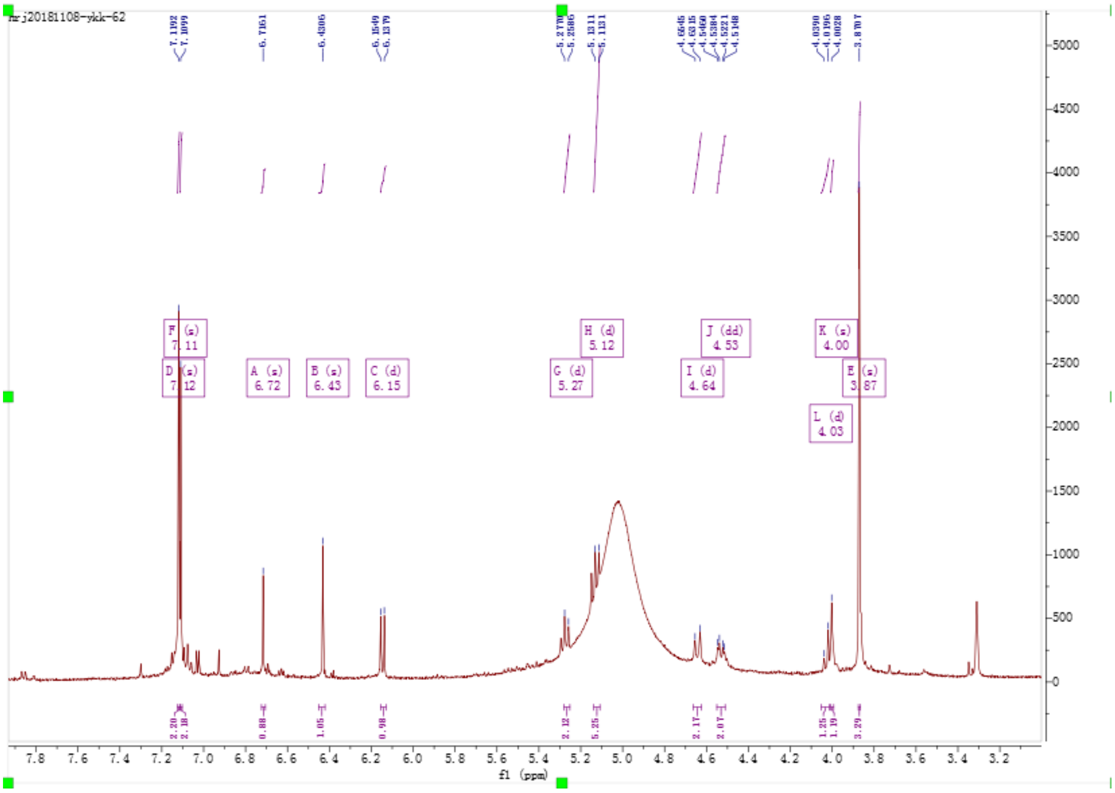

Figure S8. ^{1}H NMR (500 MHz, Methanol-d_4) spectrum of **2**

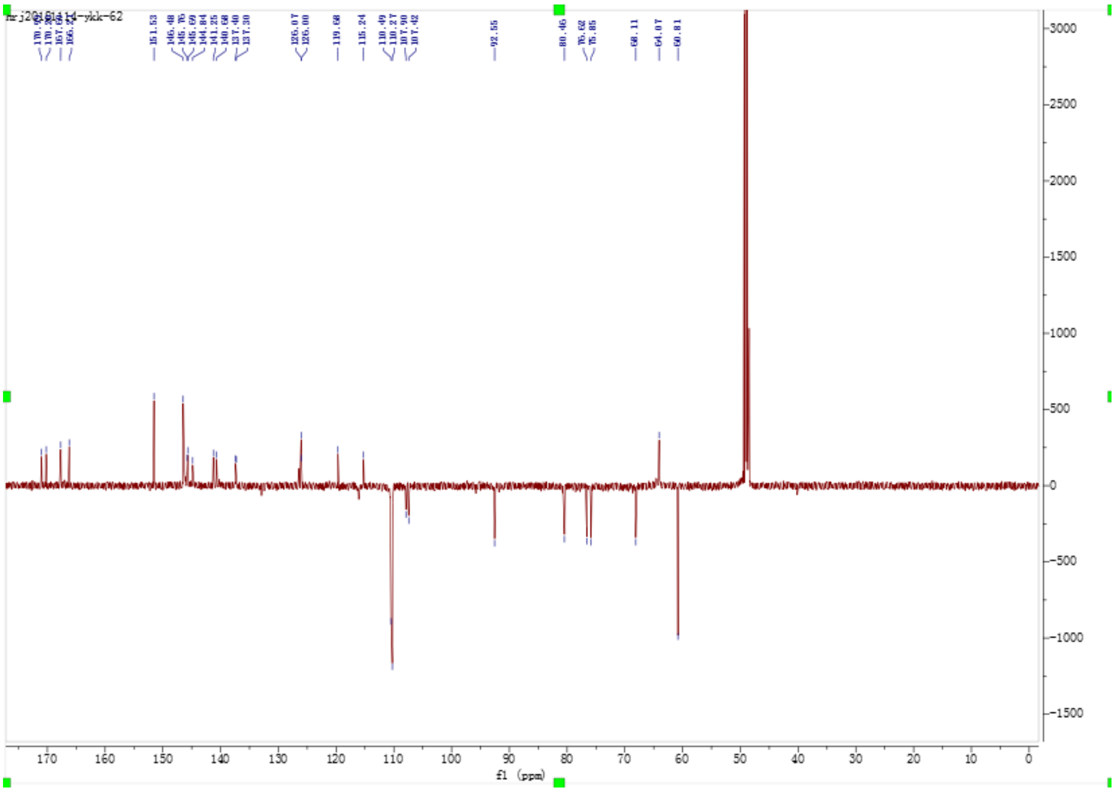

Figure S9. DEPTQ (125 MHz, Methanol-d_4) spectrum of **2**

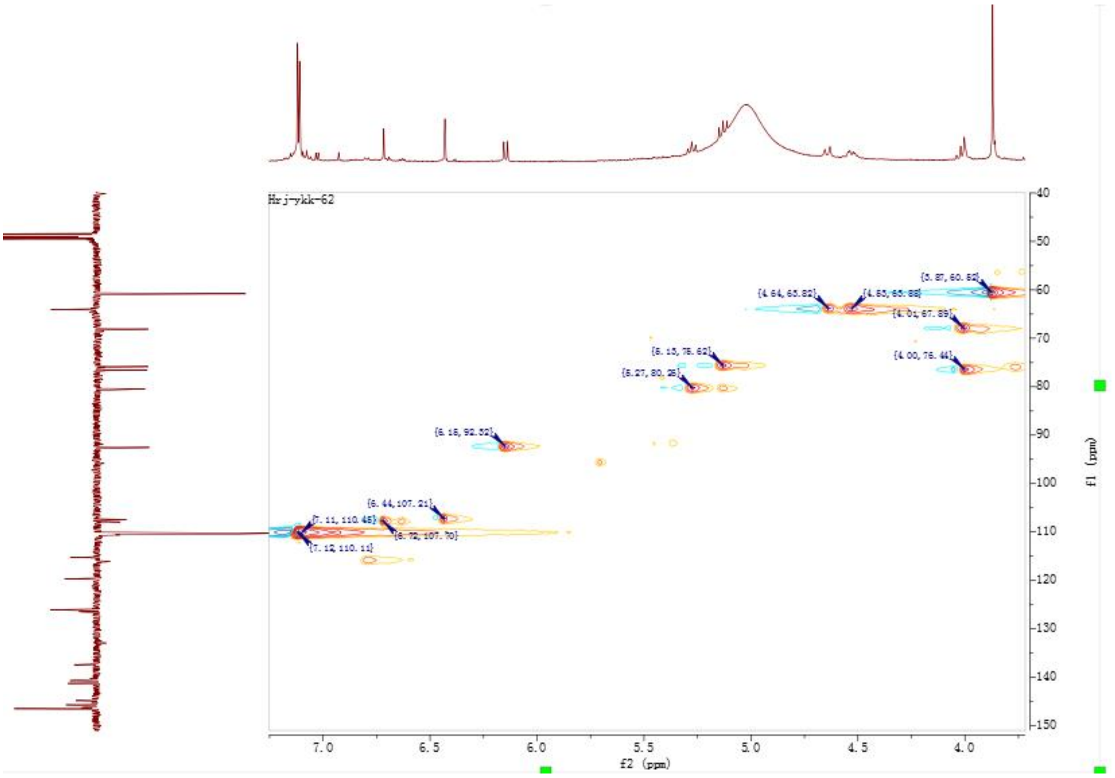

Figure S10. HSQC spectrum of **2** in Methanol-d_4

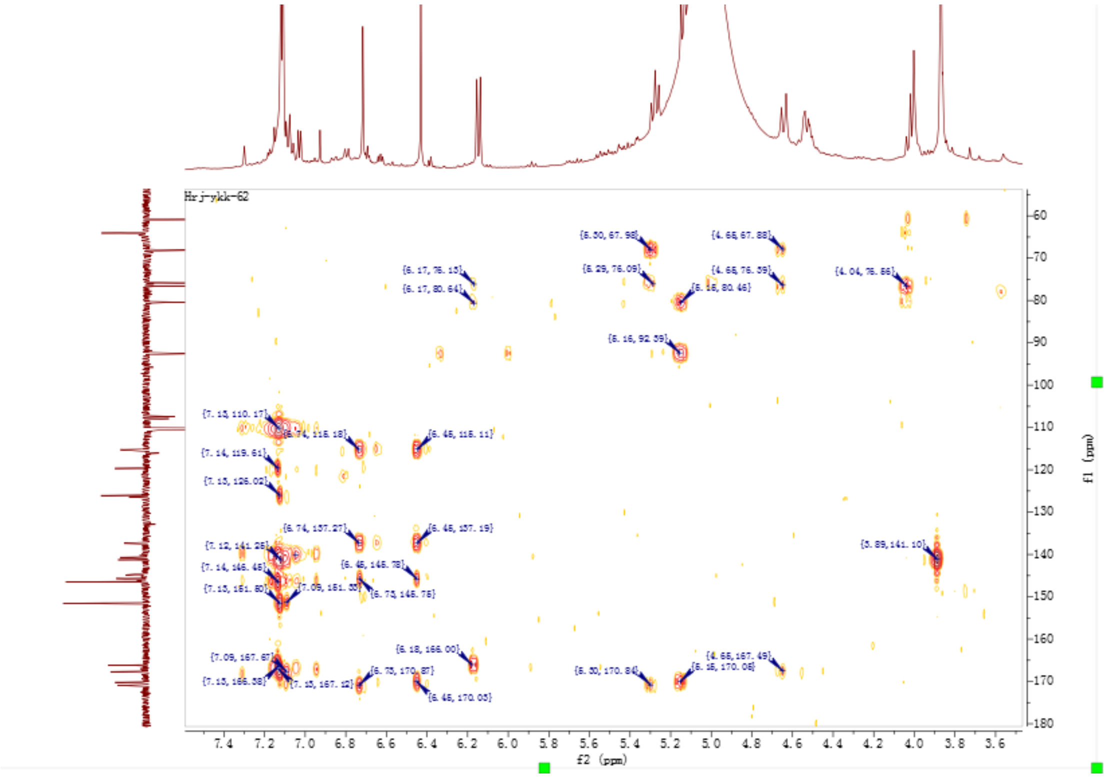

Figure S11. HMBC spectrum of **2** in Methanol-d_4

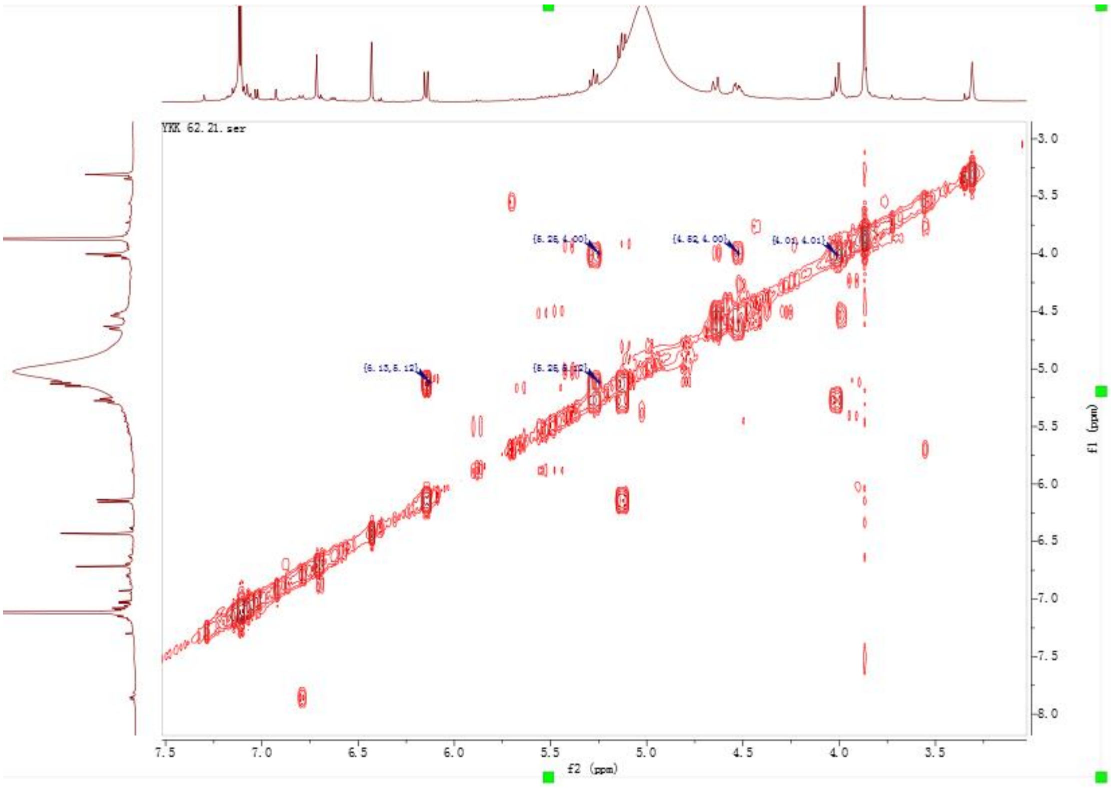

Figure S12. ^{1}H-^{1}H COSY spectrum of **2** in Methanol-d_4

Elmt	Val.	Min	Max	Elmt	Val.	Min	Max	Use Adduct
H	1	1	100	O	2	1	100	H
C	4	1	100	S	2	0	0	
N	3	0	0					

Error Margin (ppm):	50	DBE Range:	1.0 - 50.0	Electron Ions:	both
HC Ratio:	0.1 - 4.0	Apply N Rule:	no	Use MSn Info:	no
Max Isotopes:	all	Isotope RI (%):	1.00	Isotope Res:	10000
MSn Iso RI (%):	75.00	MSn Logic Mode:	AND	Max Results:	500

Event#: 4 MS(E-) Ret. Time : 12.547 Scan# : 1457

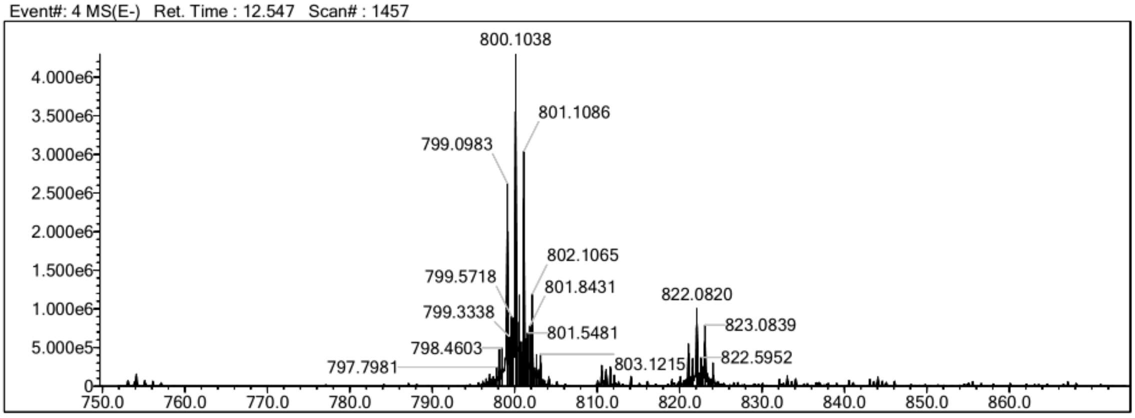

Rank	Score	Formula (M)	Ion	Meas. m/z	Pred. m/z	DBE	Df. (ppm)	Iso	Meas. (M)
1	48.75	C35 H28 O22	[M-H]-	799.0983	799.0999	22.0	-2.00	50.00	800.1056

Figure S13. HRESIMS spectrum of **2**

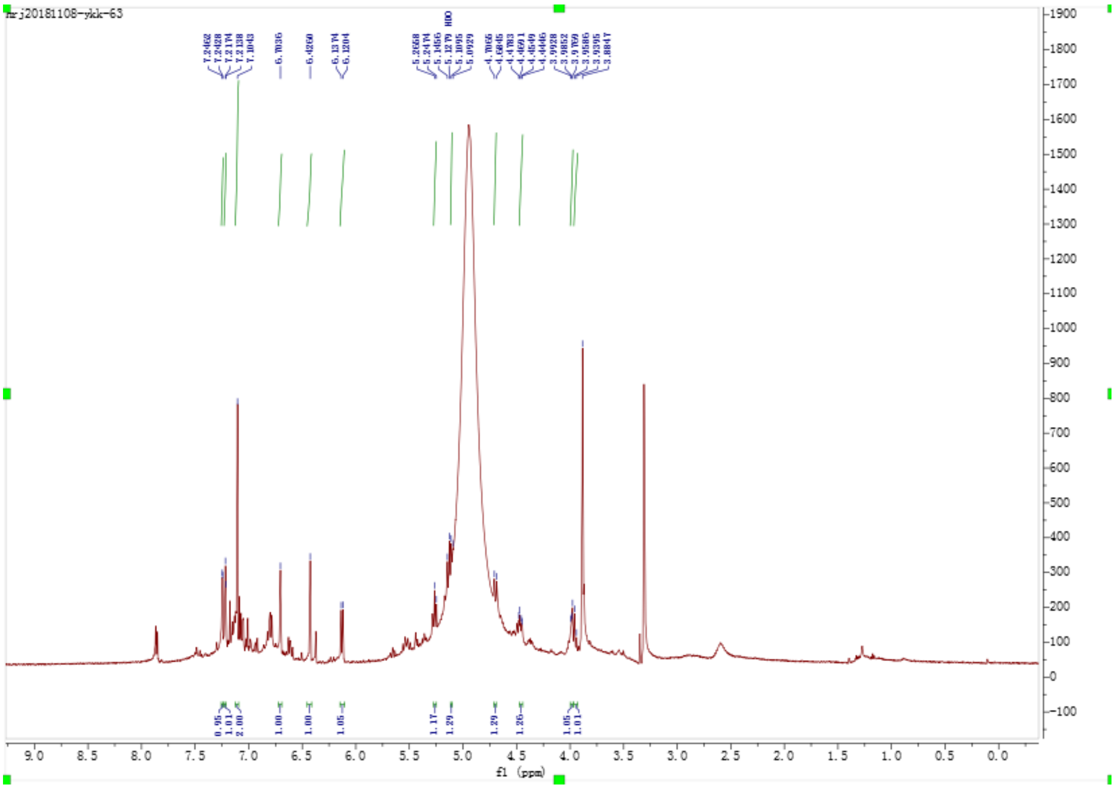

Figure S14. ^{1}H NMR (500 MHz, Methanol-d_4) spectrum of **3**

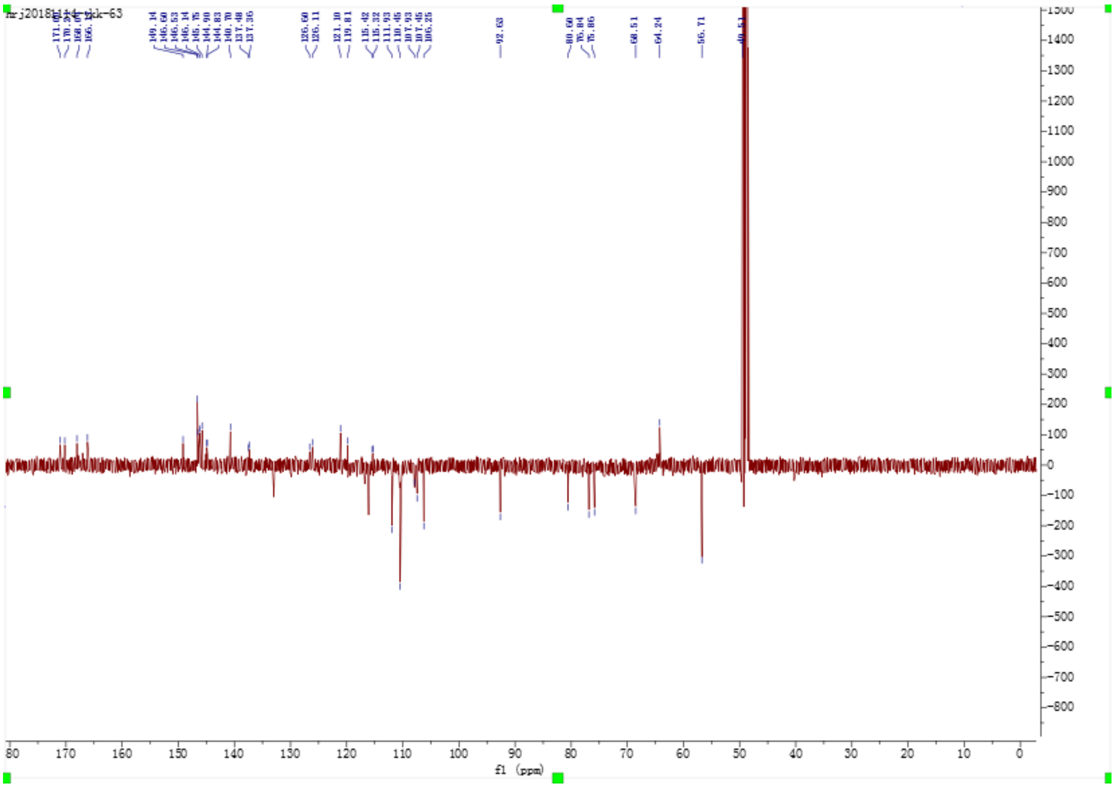

Figure S15. DEPTQ (125 MHz, Methanol-d_4) spectrum of **3**

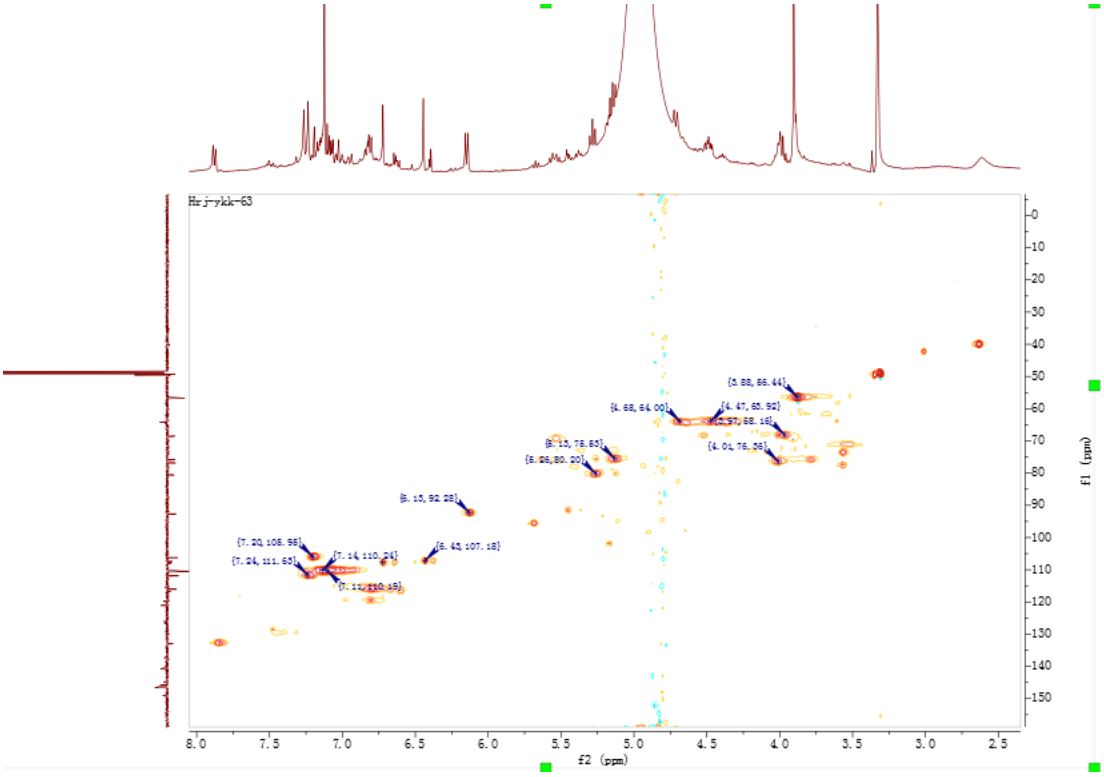

Figure S16. HSQC spectrum of **3** in Methanol-d_4

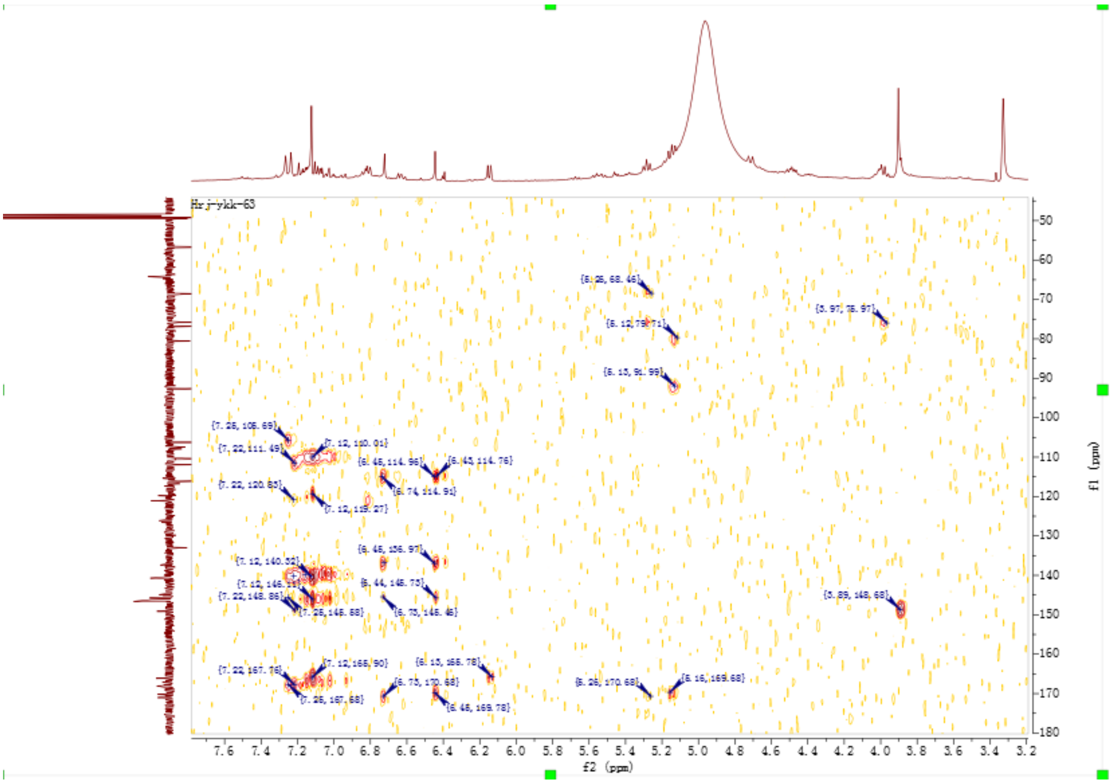

Figure S17. HMBC spectrum of **3** in Methanol-d_4

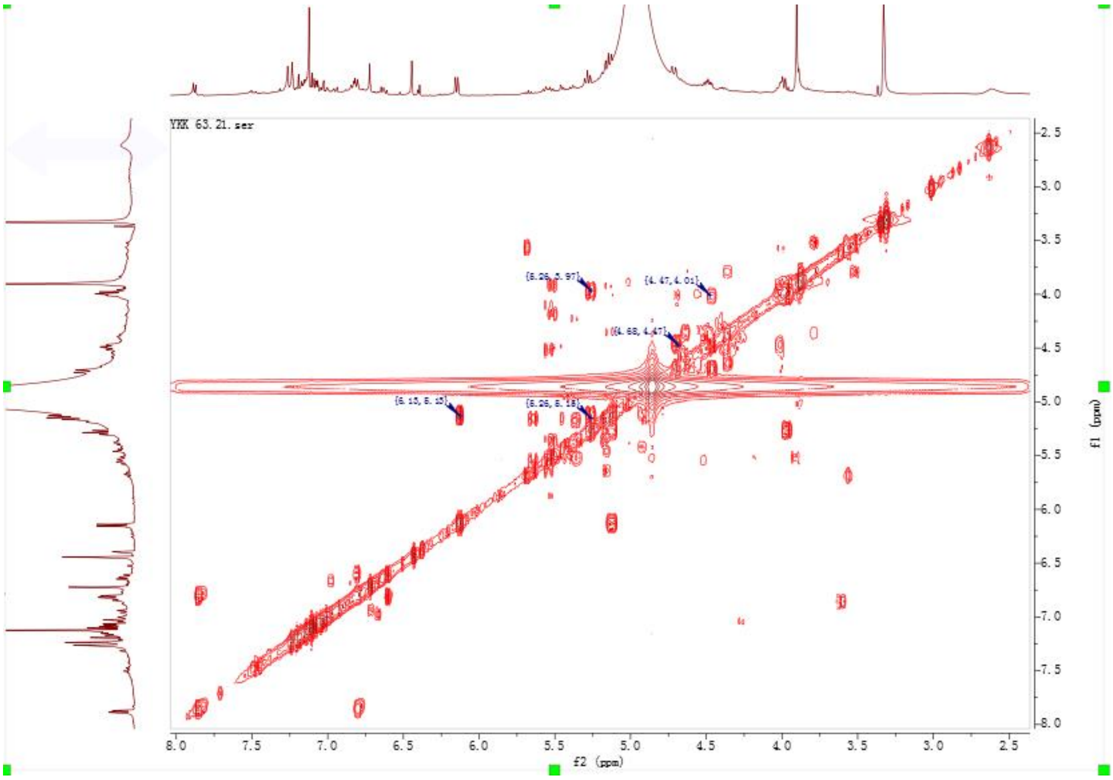

Figure S18. ^{1}H-^{1}H COSY spectrum of **3** in Methanol-d_4

Data File: F:\黄永林&何瑞杰课题组\HRJ-YKK-63.lcd

Elmt	Val.	Min	Max	Elmt	Val.	Min	Max	Use Adduct
H	1	1	100	O	2	1	100	H
C	4	1	100	S	2	0	0	
N	3	0	0					

Error Margin (ppm):	50	DBE Range:	1.0 - 50.0	Electron Ions:	both
HC Ratio:	0.1 - 4.0	Apply N Rule:	no	Use MSn Info:	no
Max Isotopes:	all	Isotope RI (%):	1.00	Isotope Res:	10000
MSn Iso RI (%):	75.00	MSn Logic Mode:	AND	Max Results:	500

Event#: 4 MS(E-) Ret. Time : 12.633 Scan# : 1474

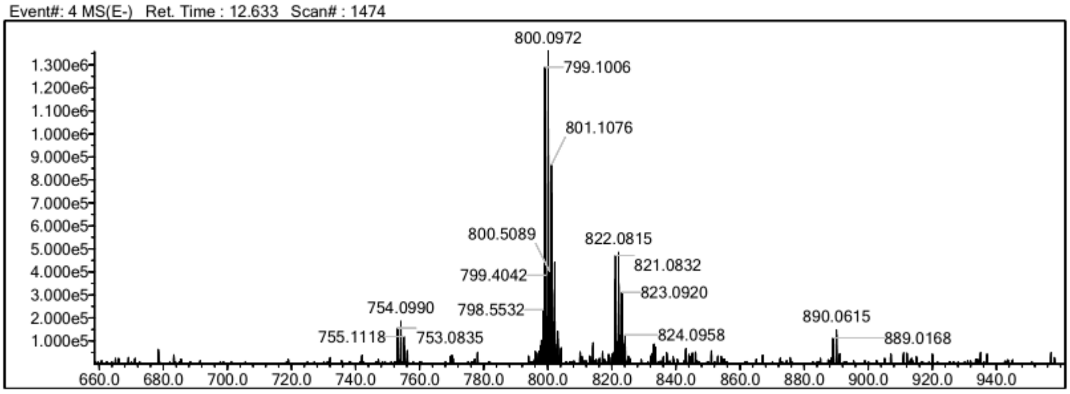

Rank	Score	Formula (M)	Ion	Meas. m/z	Pred. m/z	DBE	Df. (ppm)	Iso	Meas. (M)
1	46.30	C35 H28 O22	[M-H]-	799.1006	799.0999	22.0	0.88	46.30	800.1079

Figure S19. HRESIMS spectrum of **3**

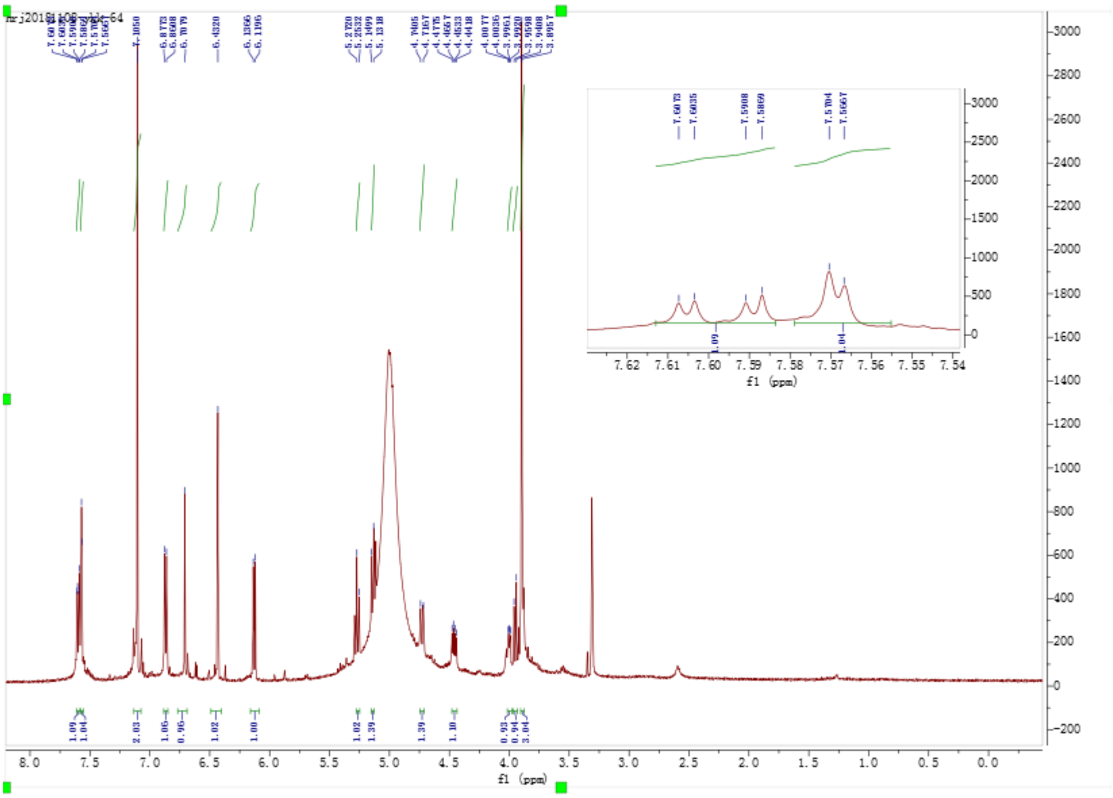

Figure S20. ^{1}H NMR (500 MHz, Methanol-d_4) spectrum of **4**

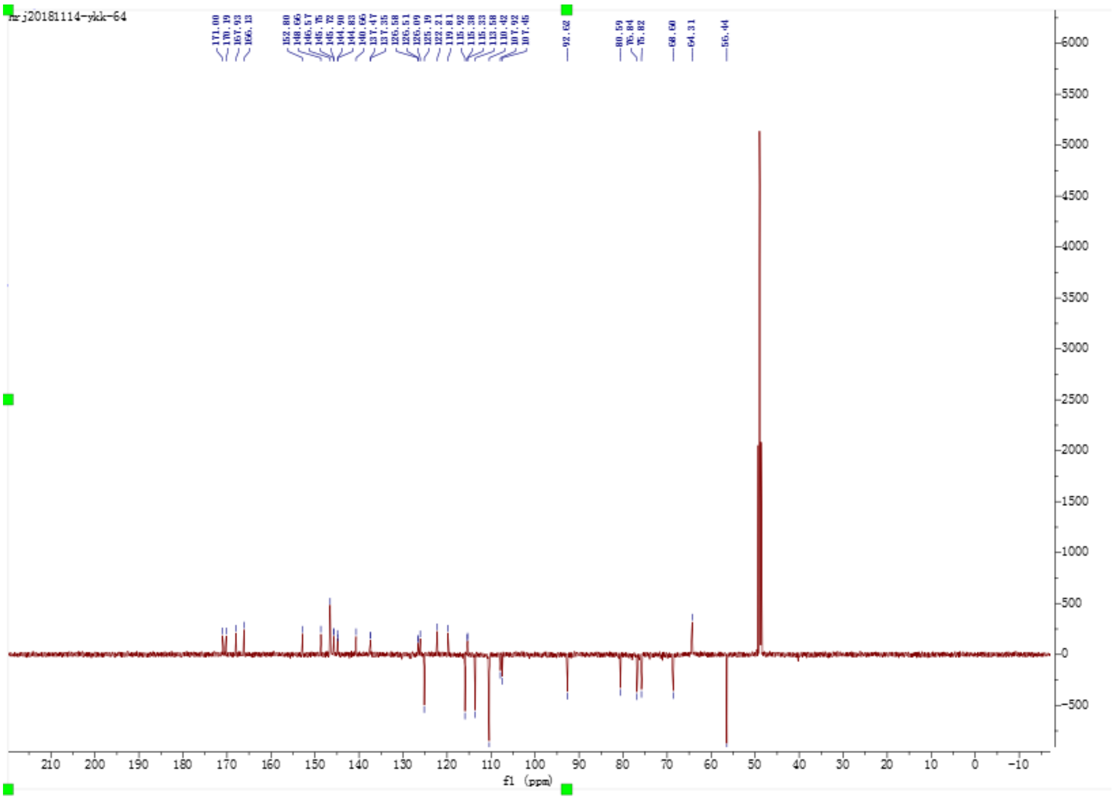

Figure S21. DEPTQ (125 MHz, Methanol-d_4) spectrum of **4**

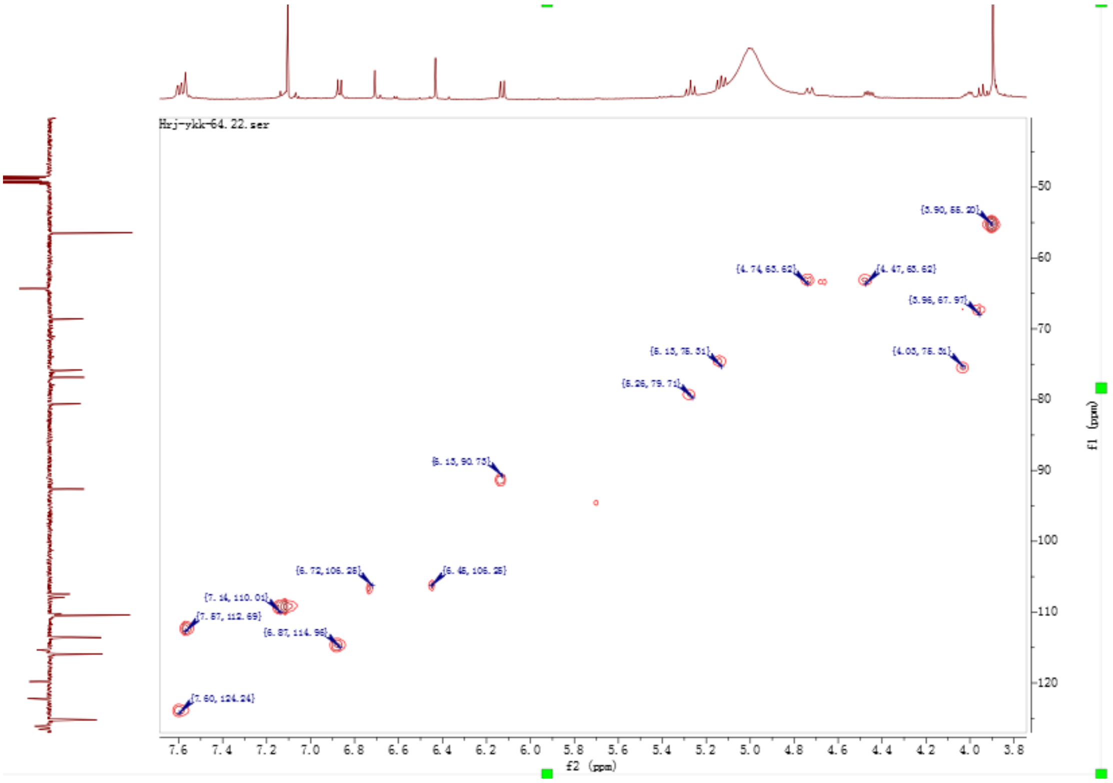

Figure S22. HSQC spectrum of **4** in Methanol-d_4

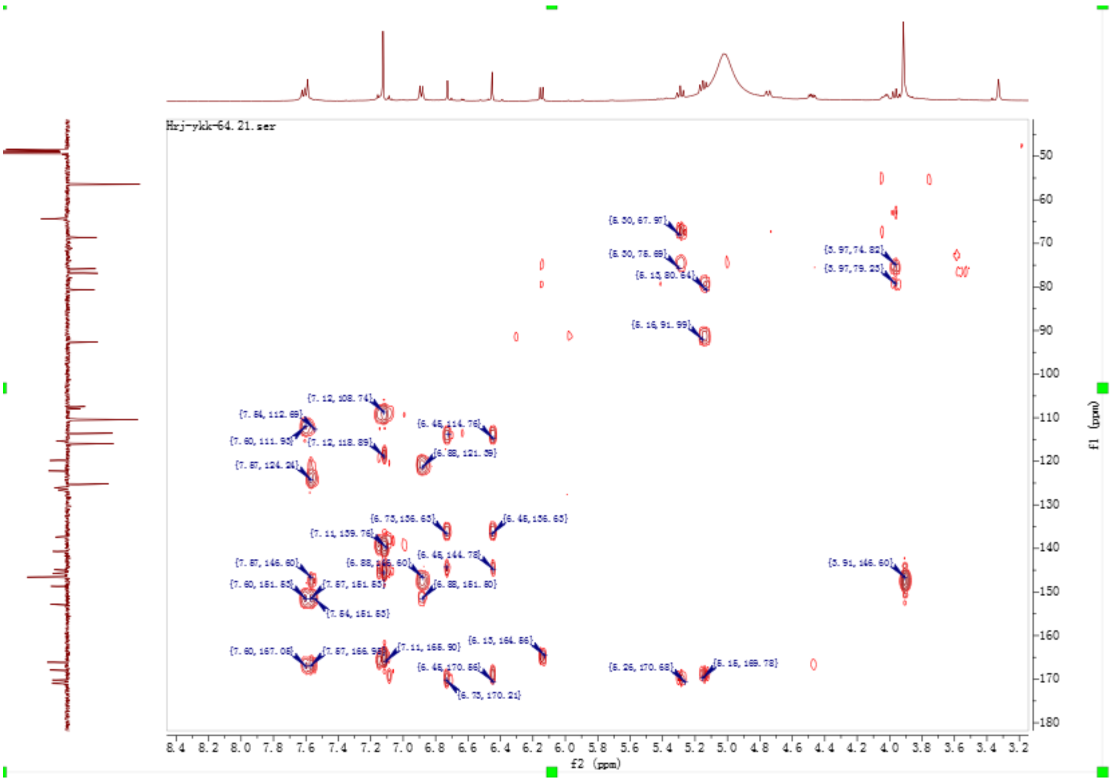

Figure S23. HMBC spectrum of **4** in Methanol-d_4

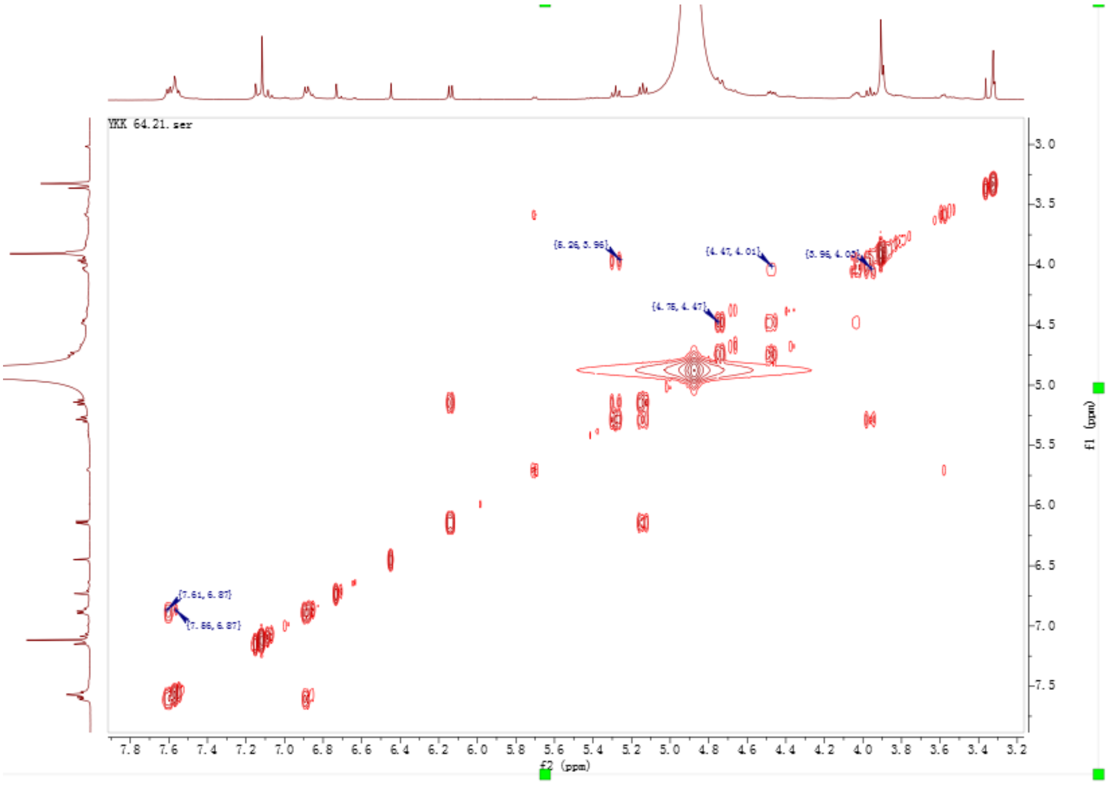

Figure S24. ^1H-^1H COSY spectrum of **4** in Methanol-d_4

Elmt	Val.	Min	Max
H	1	1	100
C	4	1	100
N	3	0	0

Elmt	Val.	Min	Max
O	2	1	100
S	2	0	0

Use Adduct
H

Error Margin (ppm): 50
HC Ratio: 0.1 - 4.0
Max Isotopes: all
MSn Iso RI (%): 75.00

DBE Range: 1.0 - 50.0
Apply N Rule: no
Isotope RI (%): 1.00
MSn Logic Mode: AND

Electron Ions: both
Use MSn Info: no
Isotope Res: 10000
Max Results: 500

Event#: 4 MS(E-) Ret. Time : 0.760 Scan# : 90

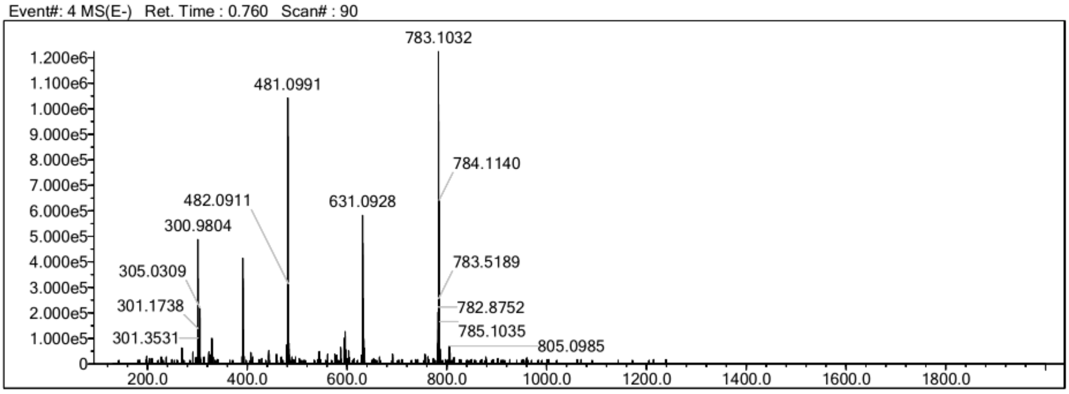

Rank	Score	Formula (M)	Ion	Meas. m/z	Pred. m/z	DBE	Df. (ppm)	Iso	Meas. (M)
2	54.38	C35 H28 O21	[M-H]-	783.1032	783.1050	22.0	-2.30	56.21	784.1105

Figure S25. HRESIMS spectrum of **4**

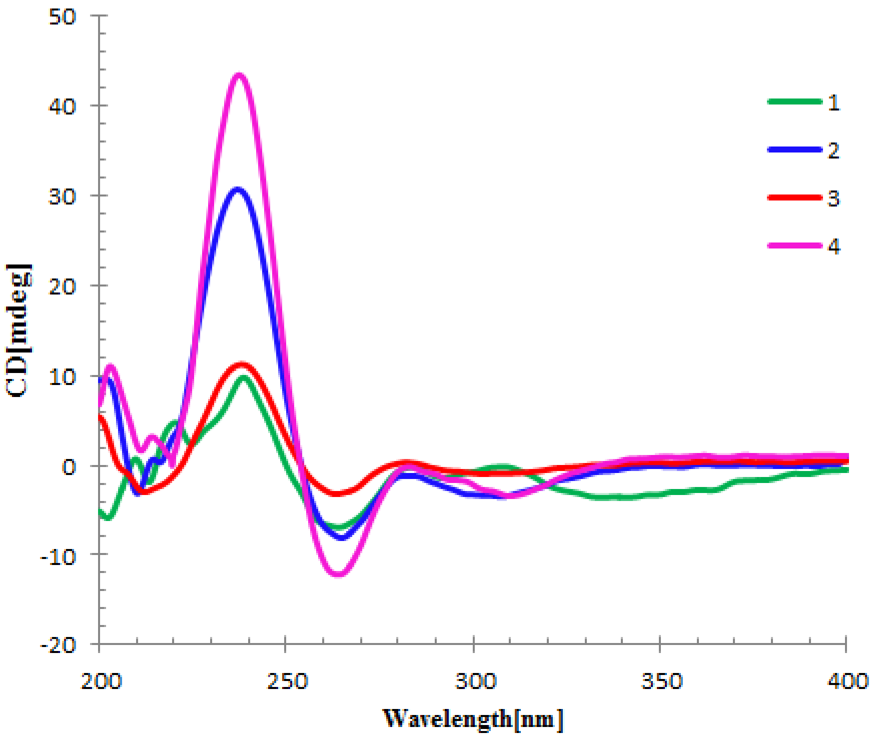

Figure S26. CD spectrum of **1**— **4**
